# Supplementary material for: Comparative genomics provides new insights into the diversity, physiology, and sexuality of the only industrially exploited tremellomycete: Phaffia rhodozyma
Source: BMC Genomics. 2016 Nov 9;17:901. doi: 10.1186/s12864-016-3244-7 (PMC5103461; doi:10.1186/s12864-016-3244-7)
Supplement: Additional file 6: — List of orphan genes with links to PFAM (related to Additional file 1: Table S1). (ZIP 1428 kb) [file 12864_2016_3244_MOESM6_ESM.zip › BLAST_HTML_FTR/G04494_P.html]

BLAST Search Results


```
BLASTP 2.2.27+


Reference:
Stephen F. Altschul, Thomas L. Madden, Alejandro A. Schäffer,
Jinghui Zhang, Zheng Zhang, Webb Miller, and David J. Lipman (1997),
"Gapped BLAST and PSI-BLAST: a new generation of protein database
search programs", Nucleic Acids Res. 25:3389-3402.


Reference for
composition-based statistics:
Alejandro A. Schäffer, L. Aravind, Thomas L. Madden, Sergei
Shavirin, John L. Spouge, Yuri I. Wolf, Eugene V. Koonin, and
Stephen F. Altschul (2001), "Improving the accuracy of PSI-BLAST
protein database searches with composition-based statistics and
other refinements", Nucleic Acids Res. 29:2994-3005.


Database: nr
           71,551,133 sequences; 26,053,659,533 total letters


Query= G04494_P

Length=123
                                                                      Score     E
Sequences producing significant alignments:                          (Bits)  Value

emb|CED83592.1|  hypothetical protein [Xanthophyllomyces dendrorh...   236    2e-77
emb|CDW72237.1|  wd repeat-containing protein 90 [Stylonychia lem...  41.6    0.093


 >emb|CED83592.1| hypothetical protein [Xanthophyllomyces dendrorhous]
Length=122

 Score =  236 bits (602),  Expect = 2e-77, Method: Compositional matrix adjust.
 Identities = 122/122 (100%), Positives = 122/122 (100%), Gaps = 0/122 (0%)

Query  1    MSLTSTTFDDQGKQPAQTLPSDASLLPASPSEDPLPEVDESYLTHRGTSASTTQSVENIY  60
            MSLTSTTFDDQGKQPAQTLPSDASLLPASPSEDPLPEVDESYLTHRGTSASTTQSVENIY
Sbjct  1    MSLTSTTFDDQGKQPAQTLPSDASLLPASPSEDPLPEVDESYLTHRGTSASTTQSVENIY  60

Query  61   GEEAERPTYDEDIAEELGGDYDLEQVGKDYEDDEGDDGEFPELEDEQEDQQTLPNEDDDE  120
            GEEAERPTYDEDIAEELGGDYDLEQVGKDYEDDEGDDGEFPELEDEQEDQQTLPNEDDDE
Sbjct  61   GEEAERPTYDEDIAEELGGDYDLEQVGKDYEDDEGDDGEFPELEDEQEDQQTLPNEDDDE  120

Query  121  DI  122
            DI
Sbjct  121  DI  122


>emb|CDW72237.1| wd repeat-containing protein 90 [Stylonychia lemnae]
Length=1933

 Score = 41.6 bits (96),  Expect = 0.093, Method: Composition-based stats.
 Identities = 34/116 (29%), Positives = 49/116 (42%), Gaps = 13/116 (11%)

Query  14   QPAQTLPSDASLLPASPSEDPLPEVDESYLTHRGTSASTTQSVENIY------------G  61
            +P + L    SL+ A  S  P    +ES  T R  S    QS EN+             G
Sbjct  247  RPKKELKQQTSLVKAGASAKPENIKEESPQTKRPKSIIRKQSSENLANNKKVGKSVKFNG  306

Query  62   EEAERPTYDEDIAEELGGDYDLEQVGKDYEDDEGD-DGEFPELEDEQEDQQTLPNE  116
            E   +P  + D+ E  G     +Q   D ED + D +G+F E   E ++ Q  P+E
Sbjct  307  EVMRQPNLNSDLEEVKGSSSSYQQYNVDEEDLQRDIEGQFNEYRQETQNYQPTPDE  362


Lambda      K        H        a         alpha
   0.305    0.130    0.363    0.792     4.96 

Gapped
Lambda      K        H        a         alpha    sigma
   0.267   0.0410    0.140     1.90     42.6     43.6 

Effective search space used: 647263899579


  Database: nr
    Posted date:  Sep 23, 2015 12:05 AM
  Number of letters in database: 26,053,659,533
  Number of sequences in database:  71,551,133


Matrix: BLOSUM62
Gap Penalties: Existence: 11, Extension: 1
Neighboring words threshold: 11
Window for multiple hits: 40
```
